# Improvement of the oxidation stability of cobalt nanoparticles

**DOI:** 10.3762/bjnano.3.9

**Published:** 2012-01-30

**Authors:** Celin Dobbrow, Annette M Schmidt

**Affiliations:** 1 Department für Chemie, Universität zu Köln, Luxemburger Str. 116, D-50939 Köln, Germany

**Keywords:** cobalt nanoparticles, core–shell particles, isothermal oxidation, nanoscale passivation, parabolic rate constant

## Abstract

In order to enhance the resistance of cobalt nanoparticles to oxidation in air, the impact of different stabilization strategies on the isothermal oxidation of particle dispersions and powders was kinetically investigated and compared to as-prepared particle preparations. A post-synthesis treatment with different alcohols was employed, and we also investigate the influence of two different polymer shells on the oxidation process. We found a parabolic decrease of the magnetization for all particle charges, indicating that the process is dominated by a diffusion of oxygen to the cobalt core and a radial growth of the oxide layer from the particle surface to the core. A significant deceleration of the oxidation process was observed for all alcohol-passivated particle preparations, and this resulted finally in a stagnation effect. The stabilizing effect increases in the sequence Co@OA/MeOH < Co@OA/EtOH < Co@OA/iPrOH. For polymer-coated particle preparations Co@PCL and Co@PS, the deceleration was even more pronounced. The results demonstrate that cobalt nanoparticles can effectively be protected against oxidation in order to improve their mid- to longterm stability.

## Findings

Magnetic nanoparticles are currently given great attention due to their application potential in data storage [1] and in sensor applications [2], as well as for biomedical uses in therapy and diagnosis. By opening novel mechanisms for drug targeting [3], controlled drug release, hyperthermia [4–5] and imaging applications [6–7] there is a need for well-controlled magnetic particle dispersions with strong magnetic properties and a good stability against oxygen and water. With a high saturation magnetization and strong magnetic anisotropy, cobalt nanoparticles in a size range between 10 and 40 nm behave as ferromagnetically blocked, single-domain magnetic dipoles. Depending on the crystal properties, which may range from cubic to epsilon-Co modification or mixed forms, they show excellent magnetic control and high magnetic heatability and are therefore of great interest for data storage as well as for medical applications. However, a knock-out criterion is their poor stability against oxidation and hydrolysis into harmful and toxic components, and their medical applicability is thus strongly limited.

Depending on the synthetic pathway, nanoscopic cobalt crystals are sensitive to slow oxidation under ambient conditions. Different strategies have been developed in order to improve the long-term oxidation stability, including the establishment of a passivating aluminum oxide shell [8] or a protective silica [9] or gold layer [10] on the particle surface. However, these postmodification methods are often laborious, and the formation of multicore particle aggregates is often observed.

In this report, two simple and straightforward methods are investigated to improve the oxidation stability of cobalt nanoparticles. In the first method, the surface treatment of the particles with different alcohols is investigated as a strategy to impede the access to the cobalt core by molecular oxygen. In the second, the particles are modified by different polymer shells in order to achieve a stabilizing effect. Although the stabilization mechanism of the alcohol treatment is still under investigation, we nevertheless gain some initial ideas about the influence of the size and properties of the stabilizer molecule on the observed deceleration, and finally stagnation, of the isothermal oxidation process in air.

The particles in this study were synthesized by thermolysis of dicobalt octacarbonyl (Co_2_(CO)_8_) in the presence of a fatty acid (oleic acid or ricinolic acid) in dichlorobenzene [11–13]. Alcohol treatment was conducted by redispersing the purified particles in methanol, ethanol, or 2-propanol (all traces of water were removed prior to the reaction) and refluxing for 30 min, resulting in the particle preparations Co@OA/MeOH, Co@OA/EtOH, and Co@OA/iPrOH, respectively.

Cobalt nanoparticles with a brush-like shell of linear polycaprolactone (Co@PCL) were obtained by a ring-opening polymerization process of ε-caprolactone starting from ricinolic acid-capped cobalt [13]. Polystyrene coated particles (Co@PS) were accessed by replacing the fatty acid stabilizer during the thermolysis of Co_2_(CO)_8_ with carboxylic acid-telechelic polystyrene, which was obtained by atom transfer radical polymerization (ATRP) [14] (see Supporting Information File 1 for details). All synthetic steps involved were performed under argon in order to prevent premature oxidation. The average particle diameters as determined by various methods are summarized in Table 1.

**Table 1:** Properties of the cobalt particle preparations employed in this study. *d*_c_: Core diameter by TEM, *d*_M_: Magnetic diameter from χ_ini_ (VSM), *d*_h_: Number-averaged hydrodynamic diameter (DLS), *M*: Molar mass of the stabilizer OA, PCL or PS.

sample	*d*_c_ (nm)	*d*_M_ (nm)	*d*_h_ (nm)	*M* (g∙mol^−1^)

Co@OA	11.9	9.1	10.1	282
Co@PCL	12.0	10.3	16.2	4108
Co@PS	17.5	13.6	25.8	6102

**Figure 1:**
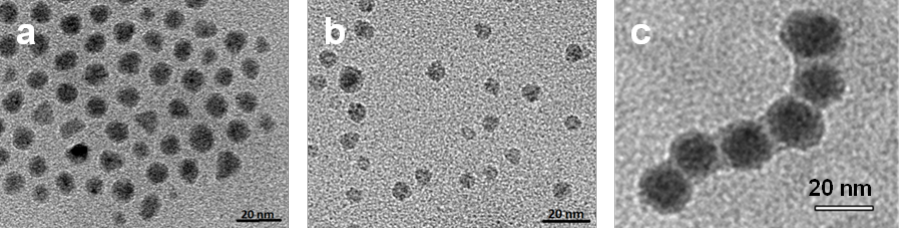
TEM images of (a) Co@OA, (b) Co@PCL, (c) Co@PS particles.

All particle species exhibit a spherical shape in transmission electron microscopy (TEM) images with a moderate size distribution (Figure 1). The volume-average magnetic core diameter *d*_M_ as calculated from the initial susceptibility χ_ini_ is in all cases lower than the average diameter *d*_c_ as determined from TEM.

In order to investigate the isothermal oxidation process, all particle species were redispersed in toluene to a concentration of 0.5 wt %, and stored in air at 25 °C. Samples were taken at different points in time up to 20 days from synthesis, and were immediately investigated with respect to their magnetic properties by vibrating sample magnetometry (VSM). In addition to the dispersions, the dried powders of selected preparations were investigated.

**Figure 2:**
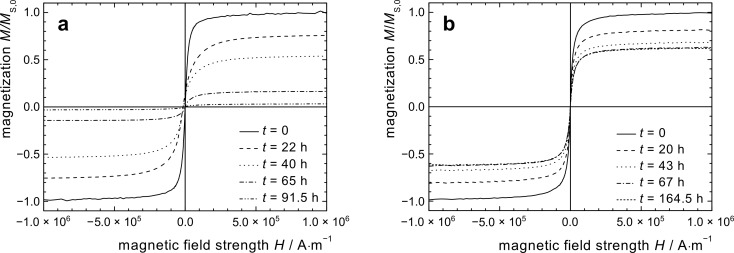
Magnetization graphs of toluene-based particle dispersions sampled at selected points of storage time at 25 °C in air, normalized by the saturation magnetization *M*_s,0_ at *t* = 0. (a) Co@OA, b) Co@OA/iPrOH.

The magnetization curves of all toluene-based particle charges show a Langevin-like behavior that is characterized by an S-shaped course of the magnetization with the applied field strength and virtually no hysteresis (see below in Figure 2, Figure 5 and Figure 6) [15]. This is in accordance with an ensemble of monodomain magnetic particles finely dispersed in a good dispersant. By following the isothermal decay kinetics of the magnetic properties under standardized conditions, we extract information on the kinetic constants concerning the oxidation process, and the resulting long-term magnetic susceptibility of the particles in dispersion as well as in powder.

We investigate the course of the saturation magnetization *M*_s_ as well as of the initial susceptibility χ_ini_ with time. While *M*_s_ gives information on the residual content of magnetic material in the sample, χ_ini_ is a measure of the average magnetic moment involved in the remagnetization process for the case of monodomain particles. The saturation magnetization *M*_s_ of the magnetic dispersions give information on the concentration of the magnetic material. As it is known from previous reports, the oxidation of nanosized cobalt results in a distinct CoO phase that contains a significant portion of metastable wurtzite-type CoO. While CoO of high rock-salt-type crystallinity displays weak antiferromagnetic properties at temperatures below 16 °C, we reasonably assume that the oxide does not significantly contribute to the room-temperature magnetic properties of the samples investigated, and we deduce that the magnetic susceptibility results from the residual cobalt phase only [16]. Therefore, the conversion of the oxidation process can be extracted directly from the *M*_s_ of the sample as compared to the initial *M*_s_ value.

The normalized initial susceptibility χ_ini_

[1]
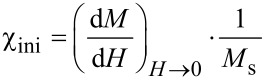


serves as an estimate for the development of the magnetic core size with time and is similarly compared to the value obtained at the begin of the process. Both values are recorded as a function of time for as-prepared fatty-acid stabilized cobalt particles as well as for surface-modified samples in toluene dispersion and for powder samples.

### Oleic acid coated particles (Co@OA)

**Figure 3:**
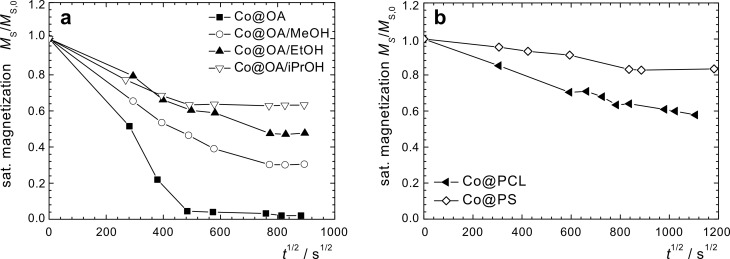
Relative saturation magnetization *M*_s_/*M_s_*_,0_ of toluene-based particle dispersions sampled as a function of time; (a) as-prepared Co@OA, and Co@OA particles treated with different alcohols; (b) polymer-coated particles Co@PCL and Co@PS.

When as-synthesized oleic or ricinolic acid coated cobalt particles were exposed to air after synthesis, a rapid decay of the magnetic properties was observed. Within the first 70 h, almost a complete loss of the magnetization took place. (Figure 2a, Figure 3a). The decay is attributed to the progressing oxidation process, which results in consumption of the magnetic cobalt phase.

The results show that the relative saturation magnetization, *M*_s_/*M*_s,0_ decreases parabolically with time *t*, according to

[2]
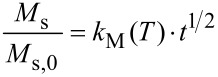


where *k*_M_(*T*) is the parabolic rate constant at a given temperature *T*. For Co@OA dispersed in toluene, the experimental data agrees reasonably well with Equation 1 up to almost the full loss of magnetization. The extracted value for *k*_M_(*T* = 25 °C) amounts to 2.00 × 10^−3^ s^−1/2^ (Table 2). The parabolic rate law indicates that the oxidation process is controlled by an oxygen diffusion process across the oxidized surface layer [17].

**Table 2:** Kinetic results on the particle oxidation process as derived from VSM experiments. *k*_M_(*T*) and *k*_c_(*T*) are the parabolic rate constants at *T* (25 °C) as extracted from the *M*_s_ and χ_ini_ data, respectively; *M*_s,∞_/*M*_s,0_ is the residual saturation magnetization after stagnation; χ_ini,∞_/χ_ini,0_ is the residual initial susceptibility after stagnation.

	*toluene dispersion*				*powder*			

sample	*k_M_*(T)	*M*_s,∞_/*M*_s,0_	*k*_χ_(T)	χ_ini,∞_/χ_ini,0_	*k_M_*(T)	*M*_s,∞_/*M*_s,0_	*k*_χ_(T)	χ_ini,∞_/χ_ini,0_
	10^−3^ s^−1/2^	%	10^−3^ s^−1/2^	%	10^−3^ s^−1/2^	%	10^−3^ s^−1/2^	%

Co@OA	2.00	(2)	1.94	(2)	2.00	(2)	1.97	(2)
Co@OA/MeOH	0.92	30	n. d.	n. d.	n. d.	n. d.	n. d.	n. d.
Co@OA/EtOH	0.69	48	n. d.	n. d.	n. d.	n. d.	n. d.	n. d.
Co@OA/iPrOH	0.76	63	0.98	45	n. d.	n. d.	n. d.	n. d.
Co@PCL	0.38	n. d.	0.51	n. d.	0.53	n. d.	0.64	n. d.
Co@PS	0.20	82	0.30	68	0.29	75	0.22	88

**Figure 4:**
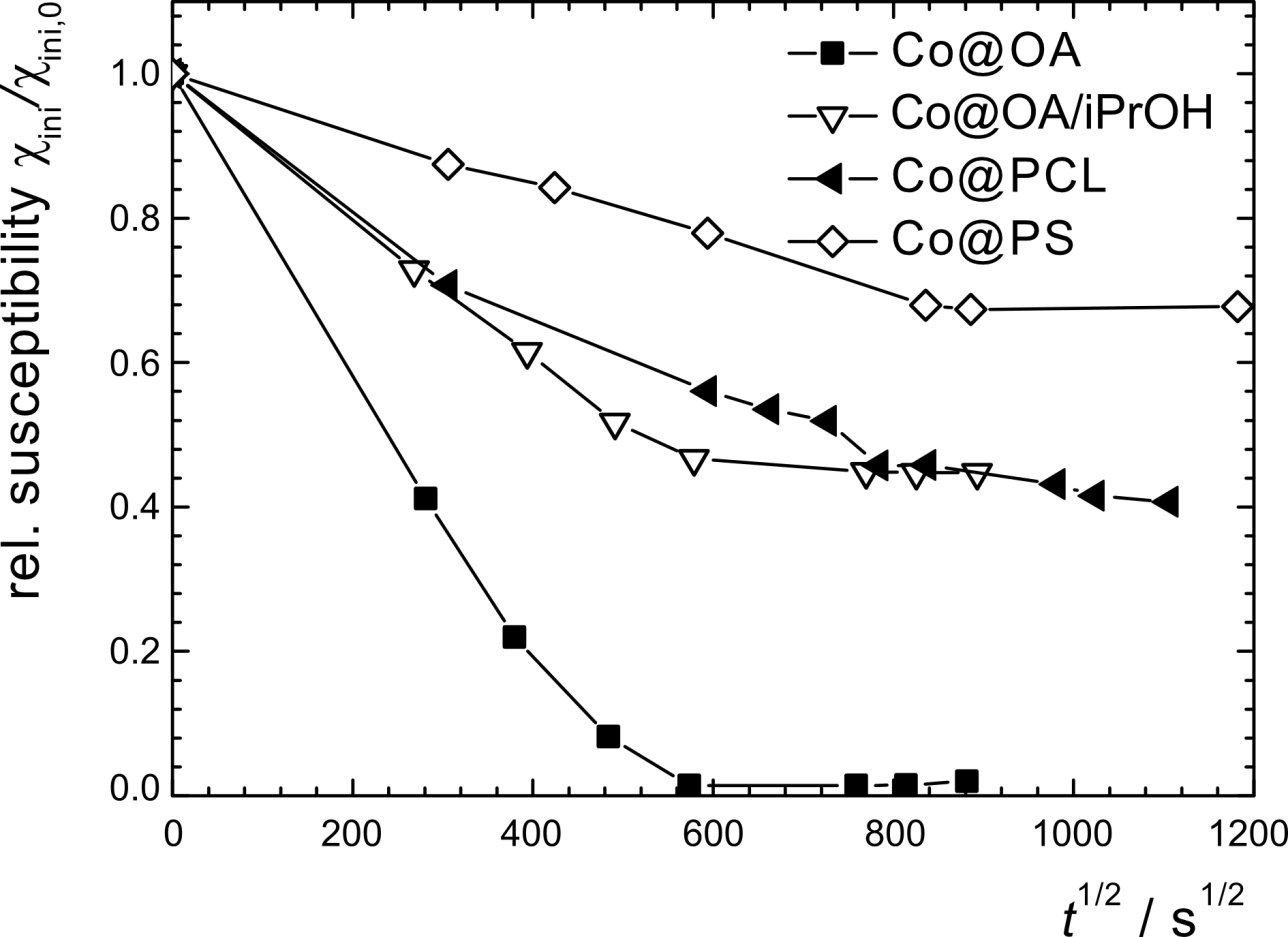
Relative initial susceptibility χ_ini_/χ_ini,0_ of toluene-based particle dispersions sampled as a function of time for as-prepared Co@OA particles, Co@OA/iPrOH particles passivated with 2-propanol, and polymer-coated particles Co@PCL and Co@PS.

From the initial susceptibility χ_ini_/χ_ini,0_ of the quasi-static magnetization curves we observe a similar trend as for *M*_s_/*M*_s,0_ (Figure 4), however, there is a significant deviation from the linear behavior for high magnetization-loss values. For early reaction times, the decay occurs on a similar scale as for the *M*_s_/*M*_s,0_ values, in agreement with the presumption that the oxidation proceeds radially from the particle surface towards the center.

### Alcohol-passivated cobalt nanoparticles (Co@OA/MeOH, Co@OA/EtOH, Co@OA/iPrOH)

All alcohol-treated particle preparations indicate an improved resistivity against oxidation by air at ambient temperature, as is obvious from the exemplary magnetization graphs shown for Co@OA/iPrOH (Figure 2b). Regarding the relative saturation magnetization *M_s_*/*M_s_*_,0_, we observe a similar parabolic decrease with time as for the untreated Co@OA samples; however, the isothermal reaction constant *k*_M_ (*T* = 25 °C) decreases strongly in the order of Co@OA > Co@OA/MeOH > Co@OA/EtOH > Co@OA/iPrOH (Table 2). In addition, the oxidation process stagnates at a constant level, which increases in the named sequence, in contrast to the behavior of the untreated samples, which show full magnetization loss. Most distinctly, Co@OA/iPrOH shows an effective stabilization against further oxidation at a level of about 63 % of the original saturation magnetization (Figure 3a).

The decrease of the initial susceptibility χ_ini_/χ_ini,0_ of Co@OA/iPrOH seems to occur slightly faster than that of *M*_s_/*M*_s,0_, as can be seen from the corresponding *k*_χ_(*T*) value. The residual value of χ_ini_/χ_ini,0_ is, with 45%, also lower than that for *M*_s_/*M*_s,0_. These observations indicate that bigger particles tend to oxidize faster than smaller particles for the alcohol-treated samples. In order to gain insight into the influence of the size of the stabilizing agent, and thus the shell thickness, and to explore the possibility of employing polymer shells as an oxygen diffusion barrier, we subjected two species of cobalt core/polymer shell particles to an analogous investigation of oxidation kinetics.

### Polycaprolactone coated cobalt nanoparticles (Co@PCL)

**Figure 5:**
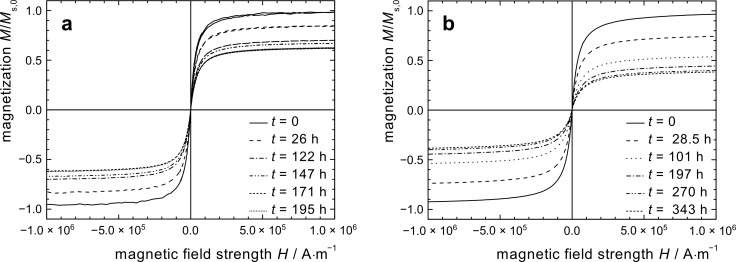
Magnetization graphs of Co@PCL particles at selected points of storage time at 25 °C in air, normalized by the saturation magnetization *M*_s,0_ at *t* = 0; (a) toluene-based Co@PCL dispersion; (b) Co@PCL particles as a powder.

Co@PCL particles employed in this study were prepared according to a recently published method from ricinolic acid coated cobalt nanoparticles by surface-initiated ring-opening polymerization of CL [13,18–19]. This results in brush-like polymer shells. Co@PCL particle oxidation in air was investigated both with a toluene-based dispersion and with a powder sample (Figure 5).

The magnetization graphs of the polycaprolactone coated particles in dispersion and as powder exposed to air show no hysteresis, indicating a superparamagnetic behavior of the particles, and a decrease of the saturation magnetization with time. The values for *M*_s_/*M*_s,0_ (Figure 3b) as well as for χ_ini_/χ_ini,0_ (Figure 4) follow a parabolic behavior with time that is considerably slower than for Co@OA particles. However, in the observed period of 20 days, no stagnation of the oxidation process was observed. It can be assumed that the polymer shell has an impact on the diffusion process of oxygen towards the particle surface.

### Polystyrene coated cobalt nanoparticles (Co@PS)

**Figure 6:**
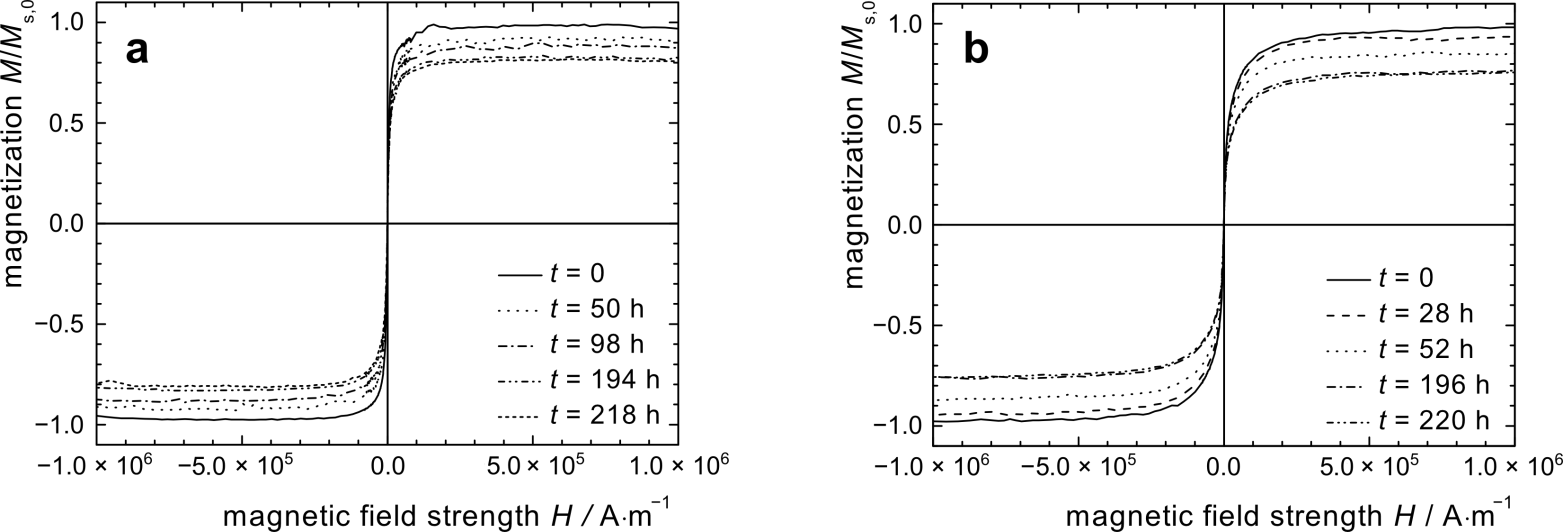
Quasi-static magnetization curves of Co@PS based particles exposed to air (a) in dispersion and (b) as powder.

The synthesis of Co based particles with a polystyrene shell was performed by thermolysis of Co_2_(CO)_8_ in the presence of carboxylic acid-telechelic polystyrene [14]. Co@PS particles were exposed to isothermal oxidation in air at 25 °C, both in toluene dispersion and as a powder. While the curves demonstrate the same general shape as already observed for other samples (Figure 6), the parabolic decrease of the saturation magnetization and the initial susceptibility with time is considerably slower (Figure 3b and Figure 4), with a reduction in the reaction constant by a factor of 10 as compared to uncoated Co@OA (Table 2).

**Figure 7:**
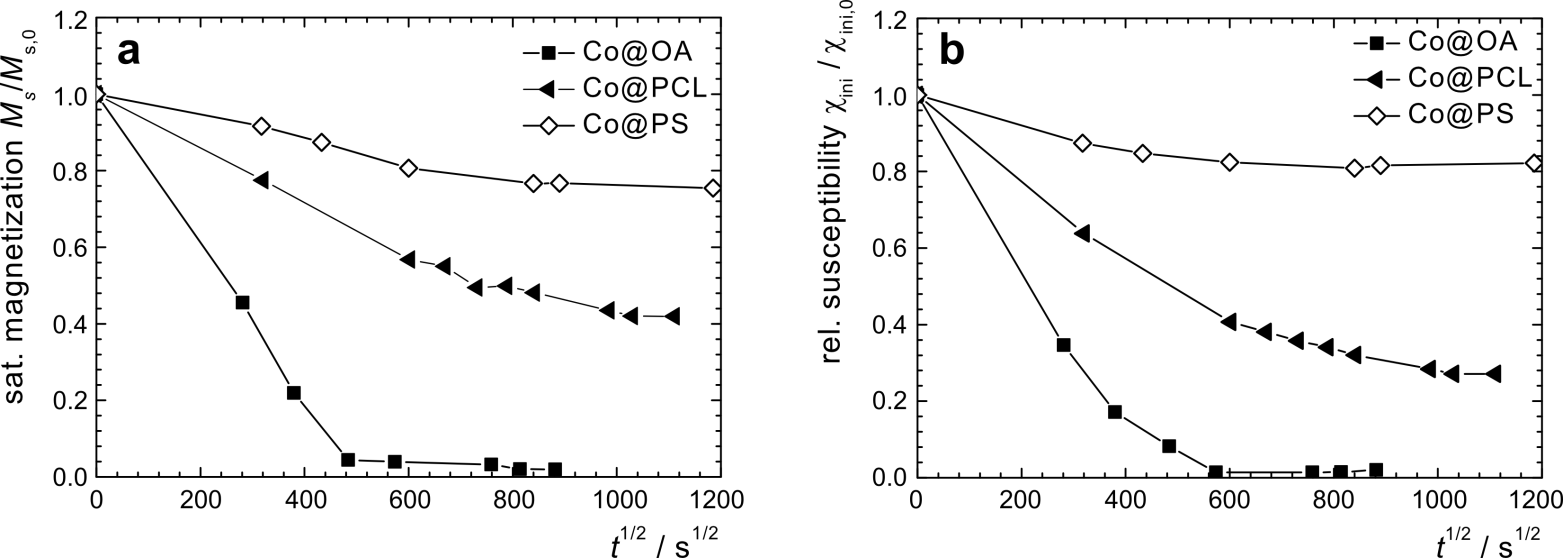
Magnetic properties of Co@PCL and Co@PS particles as a powder, as a function of storage time. (a) Relative saturation magnetization, (b) relative initial susceptibility.

The magnetization stagnates at a comparably high value of 72% (Figure 7a). Also for the long-term behavior of χ_ini_, and the respective values for the powder samples, high saturation values were obtained (Figure 7b). One possible reason for these observations is the effect of the polymer on oxygen diffusion towards the particle surface. The deceleration is much more significant for PS than for a PCL shell, probably due to a difference in their molecular oxygen diffusion properties resulting from different polymer polarity and oxygen affinity. The combination of a considerable deceleration of the oxidation progress in air, and the observed stagnation behavior is of high interest for the practical use of cobalt nanoparticles, and the stabilization mechanism will be the subject of future studies.

The results discussed above illustrate the deceleration in oxidation rate for cobalt particles that are surface treated with alcohols or coated with polymers, in comparison to the as-prepared fatty-acid coated particles. In particular, the treatment with 2-propanol and coating with a hydrophobic polymer shell, such as polystyrene, results in a significant deceleration of the oxidation process and stagnation at a reasonable level of the magnetic properties. A parabolic course of the relative saturation magnetization *M*_s_/*M*_s,0_ as well as of the relative initial susceptibility χ_ini_/χ_ini,0_ was observed, indicating that the oxidation step is dominated by a diffusion process. It can be assumed that the oxygen diffusion towards the cobalt core bypassing the oxide layer and/or the polymer shell is the critical process. This could give rise to the optimization and further development of stabilizing mechanisms against the oxidation of cobalt nanoparticles in order to allow for intensive studies of the magnetic behavior of such particles without the need for inert atmospheres or the observation of aging effects.

## Supporting Information

File 1 Magnetic properties of particle preparations and experimental section.
