# Supplementary material for: Improvement of the oxidation stability of cobalt nanoparticles
Source: Beilstein J Nanotechnol. 2012 Jan 30;3:75–81. doi: 10.3762/bjnano.3.9 (PMC3304312; doi:10.3762/bjnano.3.9)
Supplement: File 1 — Magnetic properties of particle preparations and experimental section. [file Beilstein_J_Nanotechnol-03-75-s001.pdf]

Supporting Information

for

Improvement of the oxidation stability of cobalt nanoparticles

Celin Dobbrow and Annette M. Schmidt*

Address: Department für Chemie, Universität zu Köln, Luxemburger Str. 116, D-50939 Köln, Germany

Email: Annette M. Schmidt - annette.schmidt@uni-koeln.de

* Corresponding author

Magnetic properties of particle preparations and experimental section

S1 Magnetic properties of the employed particle preparations

Table S1: Table of the magnetic properties of Co@OA, Co@PCL and Co@PS particles.

	**toluene dispersion**			**powder**		
**Sample**	M_s $A{\cdot}m^{-1}$	χ_{ini}	μ_{Co} mass %	M_s $A\,m^2kg^{-1}$	χ_{ini} m^3kg^{-1}	μ_{Co} mass %
**Co@OA**	640	0.008	0.51	32.8	0.001	20.9
**Co@PCL**	704	0.020	0.52	55.6	0.001	35.4
**Co@PS**	809	0.123	0.59	37.5	0.001	23.9

S2 Experimental part

S2.1 Materials and instrumentals

S2.1.1 Materials

Dicobalt octacarbonyl ($Co_2(CO)_8$, 95 %, 5 % hexane as stabilizer) was purchased from Sigma and stored under argon at 4 °C. 1,2-Dichlorobenzene (DCB, anhydrous, 99 %) was obtained from Aldrich. Tri-octylphosphine oxide (TOPO, 99 %, Acros Organics), ricinolic acid (85 %, TCI), oleic acid

(laboratory reagent grade, Fischer Scientific) were used without further purification. ε-Caprolactone (98 %, Merck) was dried over calcium hydride (92 %, Riedel-de-Haën) vacuum distilled, and stored under argon and molecular sieves (3 Å). Methanol (technical grade) was dried over magnesium and distilled. Ethanol (technical grade) is dried with sodium, and refluxed with diethylphthalate, followed by a vacuum distillation. Toluene was dried over sodium, vacuum distilled and stored on molecular sieves (3 Å) under argon. Acetone (technical grade) was dried with phosphorous pentoxide (98 %, Acros Organics), distilled and stored on molecular sieves (3 Å) under argon. α-Bromoisobutyric acid, 2,2-bipyridyl (bipy) and dimethylformamide (DMF) were purchased from Aldrich and were used as received. Copper (I) bromide (CuBr) was obtained from Aldrich and purified by stirring in glacial acid overnight. Styrene was purchased from Aldrich and was purified in a neutral alumina column.

S2.1.2 Instrumental

Quasi-static magnetometry was implemented on an ADE Magnetics Vibrating Sample Magnetometer (VSM) EV7. Dynamic Light Scattering (DLS) measurements were performed on a High-Performance Particle Sizer HPP5002 (Malvern Instruments) in toluene at 25 °C, with 1 x 1 cm^2 quartz glass cuvettes. Size Exclusion Chromatography (SEC) was performed in tetrahydrofurane (THF) from a system consisting of a column combination (Latek/styragel 50/1000 nm pore size) and a Waters/Millipore 481 UV detector. The system was calibrated versus commercially available polystyrene standards. Elemental analysis (EA) of carefully washed and dried particles was carried out with an Elementar Vario EL analyzer. Transmission Electron Microscopy (TEM) images were obtained with a Zeiss LEO 912 Omega, Oberkochen, Germany from samples prepared by drop-casting on a carbon-coated copper grid.

S2.2. Synthesis

All synthetic procedures were performed under an argon atmosphere.

S2.2.1. Synthesis of fatty acid coated cobalt nanoparticles [1-3] (Co@OA, Co@RA)

The synthesis of carboxylic acid coated cobalt nanoparticles was performed by thermolysis in the presence of a fatty acid stabilizer. 0.8 g (2.34 mmol) of $Co_2(CO)_8$ in 8 mL 1,2-dichlorobenzene was rapidly injected into a refluxing bath of 12 mL DCB in the presence of 0.4 mL (1.34 mmol) oleic acid, or 0.32 mL (1.08 mmol) ricinolic acid and 0.16 g (0.41 mmol) TOPO at 180 °C. After 30 min of heating, the reaction mixture was cooled down to room temperature. The batch of the oleic acid coated particles was split into portions of 4.0 mL, and the particles were precipitated with acetone and washed.

S2.2.2. Alcohol treatment of oleic acid coated cobalt particles (Co@OA/MeOH, Co@OA/EtOH, Co@OA/iPrOH)

For passivation experiments, five samples of purified oleic acid decorated cobalt particles (see 2.2.1.) were redispersed in 5 mL of each of the following alcohols: isopropyl alcohol, methanol, and ethanol. All samples were heated to reflux for 30 min. Afterwards the particles were redispersed in 4 mL of toluene.

S2.2.3. Synthesis of polycaprolactone coated cobalt particles (Co@PCL)

The polymerization of ε-caprolactone from cobalt nanoparticle surfaces was performed by adding 1.97 mL (17.75 mmol) of ε-caprolactone into 20 mL of a toluene-based dispersion of ricinolic acid coated cobalt particles (Co@RA, see S2.2.1). The reaction mixture was heated to 130 °C. After two hours reaction time, the colloidal dispersion was cooled down to ambient temperature. The core-shell particles were precipitated, washed with an excess of hexane, and redispersed in 20 mL of toluene. The particle dispersion was separated into portions of 6.0 mL. One portion was dried in order to examine powder oxidation in air.

S2.2.4 Synthesis of carboxylic acid-functionalized polystyrene

The polymer was prepared by Atom Transfer Radical Polymerization (ATRP), in which 0.90 g (6.28 mmol) Cu(I)Br, 1.00 g (6.08 mmol) 2-bromo-2,2-dimethyl-propionic acid, and 1.80 g (11.6 mmol) 2,2-bipyridyl (bipy) were added into a Schlenk flask filled with argon. 5 mL of degassed DMF was added and stirred until a red complex was formed. 60.0 mL (614 mmol) of styrene was added into the flask, and the mixture was placed into an oil bath at a temperature of 110 °C for 48 hours. After being allowed to cool to room temperature, the reaction mixture was diluted in 300 mL dichloromethane and passed through an alumina column to remove the copper catalyst. The obtained polymer solution was concentrated and precipitated into methanol. The polymer was vacuum dried and a white powder was obtained. The molar mass by SEC was M_n 6102 g·mol^{-1}, M_W 8232 g·mol^{-1}.

S2.2.5. Synthesis of polystyrene coated cobalt particles [3]

The preparation of cobalt particles capped with end-functionalized polystyrene was performed by thermolysis of $Co_2(CO)_8$ in the presence of carboxylic acid-functionalized polystyrene (see S2.2.4). 0.8 g (2.34 mmol) of $Co_2(CO)_8$ dissolved in 8 mL of 1,2-dichlorobenzene was rapidly injected into a refluxing bath of 12 mL 1,2-dichlorobenzene at 180 °C in the presence of 0.4 g (6.67·10^{-2} mmol) of polystyrene. After 30 min of heating, the reaction mixture was cooled down to ambient temperature. The particles were washed with ethanol several times and the obtained particles were redispersed in 20 mL toluene. The particle dispersion was separated into portions of 6 mL. One portion was dried to obtain a particle powder.

References

1. Gürler, C.; Feyen, M.; Behrens, S.; Matoussevitch, N.; Schmidt, A. M. *Polymer* **2008,** *49*, 2211–2216. doi:10.1016/j.polymer.2008.03.012
2. Pyun, J. *Polym. Rev. (Philadelphia, PA, U. S.)* **2007,** *47*, 231–263. doi: 10.1080/15583720701271294
3. Keng, P. Y.; Shim, I.; Korth, B. D.; Douglas, J. F.; Pyun, J. *ACS Nano* **2007,** *1,* 279–292. doi: 10.1021/nn7001213
